# CF-PA^2^Vtech: a cell-free human protein array technology for antibody validation against human proteins

**DOI:** 10.1038/s41598-019-55785-5

**Published:** 2019-12-18

**Authors:** Ryo Morishita, Shusei Sugiyama, Miwako Denda, Soh Tokunaga, Kohki Kido, Ryouhei Shioya, Satoshi Ozawa, Tatsuya Sawasaki

**Affiliations:** 1 CellFree Sciences. Co. Ltd., 3 Bunkyo-cho, Matsuyama, Ehime 790-8577 Japan; 2 Proteo-Science Center, 3 Bunkyo-cho, Matsuyama, Ehime 790-8577 Japan

**Keywords:** Assay systems, Blood proteins

## Abstract

Antibodies are widely used for the detection of specific molecules such as peptides, proteins, and chemical compounds. The specificity of an antibody is therefore its most important feature. However, it is very difficult to confirm antibody specificity. Recently, we made a human protein array consisting of 19,712 kinds of recombinant human proteins produced by a wheat cell-free protein production system. Here, we demonstrate a novel protein array technology for antibody validation (CF-PA^2^Vtech). Full-length human cDNAs were fused to N-terminal FLAG-GST and then synthesized by the wheat cell-free system. To construct a 20 K human protein array, about 10 to 14 kinds of human proteins were mixed and captured in each well by glutathione-conjugated magnetic beads in 12 plates or one plate with 384- or 1536-well format, respectively, using a strong magnetic device. Using this protein array plate, commercially available anti-HA or anti-PD-1 antibody reacted to 13 or three human proteins, respectively. The cross-reactivity of these proteins was also confirmed by immunoblotting. These proteins have a similar epitope, and alanine mutations of these epitope candidates dissolved the reactivity. These results indicated that CF-PA^2^Vtech is very useful for validation of antibodies against human protein.

## Introduction

An antibody molecule has three major features: 1) high specificity, 2) high affinity, and 3) a high variety of recognition molecules^1–3^. Utilizing these features, the antibody is widely used to detect specific molecules such as proteins, peptides, nucleotides, or small chemical compounds in a broad range of biotechnological applications such as ELISA (enzyme-linked immunosorbent assay), immunoblotting, immunohistochemistry, and immunoprecipitation. In addition, the monoclonal antibody (mAb) has also been used in many medical therapies such as cancer immunotherapies^4,5^ and infectious disease treatments^6–8^. In these utilizations of the antibody, especially for antibody drugs, the specificity of the antibody is the most important feature because the antibody is expected to recognize a single specific target molecule only. However, it is not easy to validate antibody specificity^9^.

Since the antibody is widely used for multiple applications as described above, the validation methods are based on these applications. For example, an antibody for detecting a specific virus should be validated by using a wide variety of related viruses to prevent false-positive reactions. According to this concept, the best validation technique for an antibody against a specific human protein requires a method using all human proteins. However, it is impossible to collect all human proteins from cells or tissues because limited proteins are expressed in them. Thus, the cross-reactivity of a general antibody is based on limited data using several kinds of extracts from selected cells and/or tissues. A simple method for antibody validation using a wide-range of human proteins would provide useful information for researchers and medical doctors.

In this study, by using the wheat cell-free system, we prepared 19,712 kinds of human proteins by N-terminal FLAG-GST fusion and subsequently developed a novel protein array technology by using 384- or 1536-well formatted magnetic plate for construction of a protein array plate to capture human proteins. The plate was used for validation of two commercially available anti-HA and anti-PD-1 antibodies, and some cross-reactive human proteins with similar epitopes were discovered. These results suggested that our approach using the human protein-array plate for antibody validation, called CF-PA^2^Vtech, is a useful method for validation of antibodies against human protein.

## Results

### Detection of antibody reaction using the CF-PA^2^Vtech system

**Figure 1:**
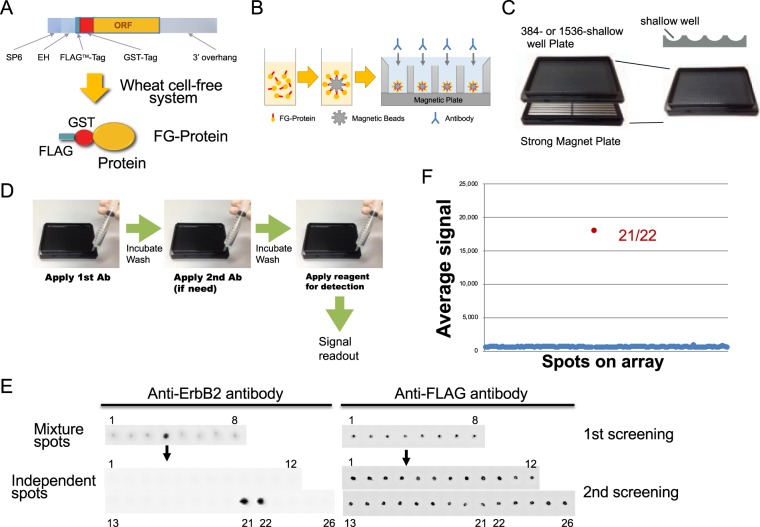
Methodology for detection of antibody reaction using CF-PA^2^Vtech system. (**A**) Schematic representation of the DNA templates and synthesized tagged proteins. N-terminal FLAG-GST (FG) tagged proteins were synthesized by the wheat cell-free system. (**B**) Structural drawing of immobilized proteins on the array. Synthesized FG-proteins were captured to the GSH magnetic beads surface. The proteins on magnetic beads were immobilized in separate wells of 384- or 1536-shallow well plate. (**C**) Structural drawing of magnetic array plate. Strong magnets were arranged under the 384- or 1536-shallow well plastic plate to immobilize the magnetic beads on the bottom of the well plate. (**D**) General assay procedure of CF-PA^2^Vtech. The first antibody is added to the array, after the reaction and the washing steps, the bonded antibodies are detected by chemiluminescence using the HRP-fused secondary antibody. (**E**) A model experiment using 104 kinds of FG-proteins on CF-PA^2^Vtech. For the first screening of anti-ErbB2 antibody targets using the array, 13-mixed proteins were placed in each well. In the second screening, the proteins in a positive spot (left and upper panel) were individually re-arrayed on a new array plate in duplicate (lower panel). Out of the 13 protein candidates, a single protein was determined as the antibody target (ERBB2 protein, 21 and 22 wells in left and lower panels). All spotted proteins were confirmed with anti-FLAG antibody (right panel). (**F**) The dot-blotting graph of signal intensity from the second screening result by CF-PA^2^Vtech. The spots showing higher signal intensity than the other spots were determined as the positive spots (ERBB2 protein shown in red spot on graph).

Previously, a human full-length cDNA set for wheat cell-free protein synthesis was reported^10^ and was used in this study. All human recombinant proteins for the array were synthesized as a fusion form of N-terminal FLAG-GST (FG) protein by a wheat cell-free protein production system (Fig. 1A) on 384-well. For construction of a protein array plate, about 14 kinds of FG-proteins were mixed with glutathione-conjugated magnetic beads, and then were washed four times with buffer to remove extra proteins from wheat embryo proteins. An automatic device was then used to capture them on a single well using a strong magnetic force (Fig. 1B,C). After a washing, 10-mL of a target antibody solution was applied and incubated for one hour. Then, the plate was washed three times with buffer (Fig. 1D). Next, the second antibody conjugating HRP (horseradish peroxidase) enzyme was applied by syringe, and then incubated for one hour. If HRP enzyme is conjugated to the target antibody used, the step using the second antibody is not required. After washing, a reagent mixture (ImmunoStar LD) was applied to detect the HRP-antibody, and subsequently a signal on the plate was read using ImageQuant LAS4000. Array-Pro Analyzer was used for signal quantification. In the case of antibodies that are fluorescence-labeled instead of HRP, Typhoon FLA 9500 was used for detection. Because each well included about 14 kinds of proteins, CF-PA^2^Vtech consists of a two-step screening, in which the first step is used to find positive mixed spot(s) and the second screening identifies individual positive clones.

For a model experiment, we used 104 kinds of FG-proteins including ERBB2 protein and two kinds of antibodies, anti-ErbB2 and anti-FLAG antibodies. This model was used to address whether the CF-PA^2^Vtech using anti-ErbB2 antibody can specifically detect ERBB2 protein. In each well, about 13 proteins were mixed and captured. According to the protocol above, the antibody reaction was carried out. After the first screening using anti-ErbB2 antibody, one single spot (fourth spot) showed a positive signal (left panel in Fig. 1E). For the second screening, 13 proteins from the single positive spot were individually observed in each well and detected by a reaction with anti-ErbB2 antibody. Using this method, a dual 21/22 spot (21 and 22) was found as a positive clone. All spotted proteins were detected by anti-FLAG antibody (right panel in Fig. 1E). The average signal was also indicated in the dot-blotting graph (Fig. 1F). As expected, the positive 21/22 spot was the ERBB2 protein. These results indicated that the CF-PA^2^Vtech system could detect the antibody reaction.

### Validation of commercially available anti-HA antibody by CF-PA^2^Vtech based on 384-well format

**Figure 2:**
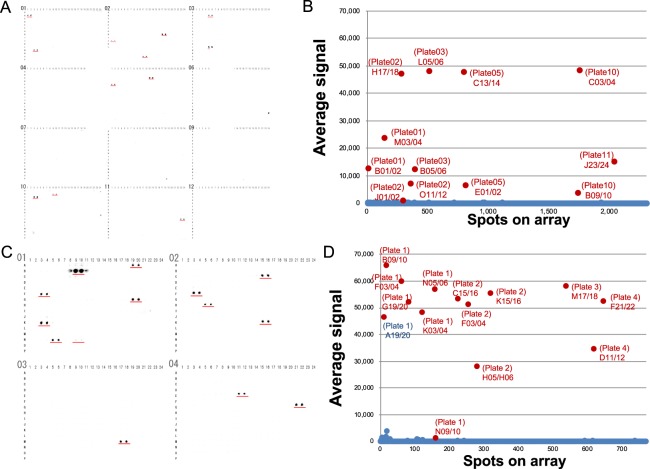
Screening of cross-reactive antigens against anti-HA antibody using CF-PA^2^Vtech (384-well plate version). (**A**) The images of the first screening using anti-HA antibody with the 384-well plate-based CF-PA^2^Vtech. In the first round of screening, using 12 plates of the 384-well plate version array in duplicate where 10 proteins are pooled per well, 12 spots were positive (red underlines). (**B**) The dot-blotting graph from the scan data of the first screening using anti-HA antibody. Average signal intensities of dual spots on the array image were plotted. On the graph, 12 positive spots showed higher signal intensities than the other spots (red colour characters). (**C**) The result images of second anti-HA antibody target screening using CF-PA^2^Vtech. The proteins in the positive result wells in the first round of screening were re-arrayed for individual testing on the array in duplicate. In the second round of screening, 13 proteins were recognized by the anti-HA-tag antibody. (**D**) The dot-blotting graph of the second anti-HA antibody target screening. Average signal intensities of dual spots of each protein on an array image were plotted. On the graph, 13 spots (red colour characters) showed higher signal intensities than the other spots and were determined as positive.

Because CF-PA^2^Vtech system could detect the antibody reaction (Fig. 1), we planned to expand the number of proteins to 19,712. For that, we thought to construct two types of protein array plates in a 384- or 1536-well plate format. For the 384-well format, 10 kinds of proteins were captured in each well as dual spots for checking reproducibility in 384 well-plates, indicating that 1,920 kinds of proteins were spotted on a 384-well plate. To cover 19,712 proteins, totally 12 plates were used for the screening (see Fig. 2A). As the first validation, commercially available anti-HA mouse mAb (TANA2 clone, MBL) was carried out using the 384-well format. Since this anti-HA mAb was conjugated to HRP enzyme directly, the signal was detected without the use of a second antibody. Like in the first screening, anti-HA-HRP mAb (1:5,000 dilution) was applied to each plate for 1 h. After washing, the plate was treated with detection mixture. The results indicated 12 dual spots as positive by imaging (Fig. 2A) and scan data (Fig. 2B). Next, for the second screening, we selected a total of 595 proteins from 12 positive spots, randomly selected 60 negative spots and control proteins including FLAG-GST (plate01 A13/14 to 17/18 and A21/22, and B13/14 to 21/22) and FLAG-GST-HA (plate01 A19/20), and then they were individually spotted in four 384-well plates as dual spots. The same reaction condition as that of the first screening was used for this second screening. The results from images (Fig. 2C) and scan data (Fig. 2D) indicated that 13 clones (red characters) reacted with anti-HA mAb (Table 1). Fortunately, all 12 positive spots from the first screening had at least one cross-reactive clone, indicating that the screening using CF-PA^2^Vtech was successful.

**Table 1:** Summary of cross-reactive clones by anti-HA antibody.

Clone no.	Plate no.	Well no.	Entry clone ID	Refeq ID	Protein	Short name	Similar epitope region	Similar epitope sequence^*^
1	1	B9/10	FLJ10511AAAF	NP_060590.1	armadillo repeat containing 1	ARMC1	233—241	QNTELPDYL
2	1	F3/4	FLJ94173AAAF	NP_004421.2	early growth response 3	EGR3	187–195	LFPMIPDYN
3	1	G19/20	FLJ40075AAAF	NP_695001.2	chromosome 20 open reading frame 96		18–26	QEFQVPDYV
4	1	K3/4	FLJ92933AAAF	NP_036514.1	tetratricopeptide repeat domain 33	TTC33	197–205	SPKSIPDYD
5	1	N5/6	FLJ20516AAAF	NP_060328.2	TIMELESS interacting protein		8–16	GVIDLPDYE
6	1	N9/10	FLJ82655AAAF	NP_981953.2	family with sequence similarity 47, member A		709–717	KKPDEPDVL
7	2	C15/16	FLJ10734AAAF	NP_060667.2	ZFP64 zinc finger protein		485–493	QPSQVPQFS
8	2	F3/4	FLJ10882AAAF	NP_071371.3	zinc finger protein 64	ZFP64	431–439	QPSQVPQFS
9	2	H5/6	FLJ81155AAAF	NP_001244031.1	NADH dehydrogenase (ubiquinone) 1 beta subcomplex, 3, 12 kDa	NDUFB3	12–20	HKMELPDYR
10	2	K15/16	FLJ31145AAAF	NP_443089.3	zinc finger protein 830		152–160	GLSLLPDYE
11	3	M17/18	FLJ54287AAAF	NP_004421.2	early growth response 3, a splice variant		28–36	TVTMIPDYN
12	4	D11/12	FLJ82936AAAF	NP_919431.2	PH domain and leucine rich repeat protein phosphatase 1		1193–1201	QLDQLPDYY
13	4	F21/22	FLJ83815AAAF	NP_001955.1	early growth response 1	EGR1	248–256	QVPMIPDYL
HA								YPYDVPDYA

^*^The amino acids identical with HA epitope sequence are shown as bold.

### Characterization of 13 human proteins reacting with anti-HA antibody

**Figure 3:**
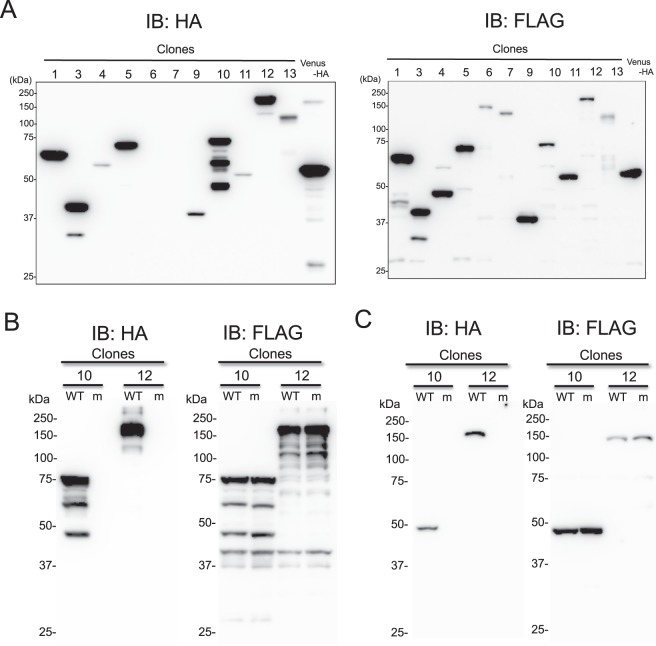
Characterization of cross-reactive antigens by anti-HA antibody. (**A**) Immunoblotting analysis of the cell-free produced proteins. These proteins were detected with anti-HA (left panel) or anti-FLAG (right panel) antibody. (**B**) Immunoblotting analysis using the cell-free produced wild (WT) and mutant (m) proteins. Clones no. 10 and 12 in Table 1 were selected for this analysis. As mutant proteins, each candidate epitope of PDY was mutated to alanine residues. Anti-HA (left panel) or anti-FLAG (right panel) antibody were used for detection of WT and m proteins. (**C**) Immunoblotting analysis using proteins expressed in HEK293T cells. The same clones in (**B**) were inserted without GST into mammalian expression vector, pcDNA3.1-FLAG, and the same antibodies in (**B**) were also used for analysis.

In Fig. 2, CF-PA^2^Vtech using anti-HA mAb indicated cross-reactivity with 13 human proteins (Table 1). From analysis of amino-acid sequences, clone no. 2 and 11, or no. 7 and 8 were splicing variants from a gene of early growth response 3 or zinc finger protein 64, respectively. Because these splicing variants have almost the same sequence, the 11 clones were used for further analysis except for clone no. 2 and 8. To confirm by immunoblotting using anti-HA and anti-FLAG antibodies, these proteins were synthesized by the cell-free system. As a result, immunoblotting using anti-HA antibody showed that 9 clones were positive except for clone no. 6 and 7 (Fig. 3A).

Next, to find the difference between reactive and non-reactive clones, we investigated whether these proteins have similar epitopes recognized by anti-HA mAb. Because YPYDVPDYA peptide has been used as an epitope for construction of anti-HA antibody, a similar epitope sequence was searched for in these clones. Interestingly, the reactive 9 proteins have a PDY as a similar epitope sequence (Table 1). To confirm whether these sequences were recognized by anti-HA mAb, we selected two clones of clone no. 10 and 12 according to the sequence difference, and subsequently each candidate epitope was mutated to the alanine sequence. These recombinant mutants were analysed by immunoblotting using anti-FLAG and anti-HA antibodies. The result showed that these mutants completely lost cross-reactivity (Fig. 3B), indicating that the proteins found by CF-PA^2^Vtech have an epitope for anti-HA mAb. Additionally, the two clones were inserted into a mammalian expression vector without the GST tag and then were expressed in HEK293T cells. The immunoblotting analysis also showed the same result (Fig. 3C), indicating that the cross-reactivity is not dependent on the expression system. The mutation analysis suggests that the anti-HA antibody we used mainly recognizes the PDY sequence as an epitope for immunoblotting after SDS-PAGE. Taken together, these results indicated that CF-PA^2^Vtech in a 384-well format is useful for identifying antigen protein epitopes of a target antibody.

### Validation of commercially available anti-PD-1 antibody by CF-PA^2^Vtech

Next, we used a protein array of a 1536-well format, in which about 14 kinds of proteins were captured on each well as a single spot, and a set consisted of a single plate. A notable feature of this format is that 19,712 human proteins were mounted on a single plate. Before screening, to investigate whether proteins could be captured on the 1536-well spot, a signal on each spot was detected by anti-FLAG antibody. Fluorescence intensity showed that more than 80% of wells were found within the high signal zone (Supplementary Fig. 1), indicating that a sufficient amount of FG-protein was captured in each well on a 1536-well array. Recently, many kinds of mAbs from rabbits have been widely used in cell biology. Thus, as an antibody for validation, we chose commercially available anti-PD-1 rabbit mAb (D4W2J, Cell Signaling Technology). Since this anti-PD-1 mAb has not been conjugated with the HRP enzyme, unlike anti-HA-HRP mAb above, a background signal from rabbit-IgG-HRP mAb (NA934, GE Healthcare) used as a second antibody was analysed before the first screening. This pre-check indicated that a single spot (17-12, green colour) was provided by the rabbit-IgG antibody (Supplementary Fig. 2), indicating that this rabbit-IgG-HRP mAb has very low cross-reactivity with human proteins.

**Figure 4:**
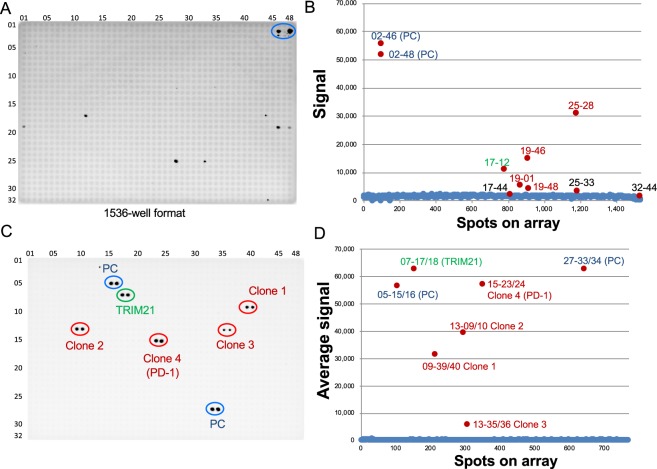
Screening results of cross-reactive antigens against anti-PD-1 antibody using CF-PA^2^Vtech (a 1536-well plate version). (**A**) The result images of the first screening using anti-PD-1 antibody by 1536-well plate based CF-PA^2^Vtech. Ten spots shown as black dots were positive. A blue ellipse indicates the spots of human PD-1 protein as a positive control. (**B**) The dot-blotting graph from scan data of the first screening using anti-PD-1 antibody. Signal intensities of each array spot were plotted. On the graph, four spots (red colour characters) were selected as positive spots. (**C**) The result images of second screening using anti-PD-1 antibody. The proteins in positive wells found in the first screening were re-arrayed in duplicate for individual testing on the array. Four proteins, shown as clone 1 to 4, were recognized by the anti-PD-1 antibody (red ellipses). Clone 4 was the PD-1 protein from the protein array. Green ellipse was TRIM21, which cross-reacts with anti-IgG antibody. (**D**) The dot-blotting graph from scan data of the second screening using anti-PD-1 antibody. Average signal intensities of dual spots on an array image were plotted. On the graph, four spots showed higher signal intensities. The two spots (blue colour character, 05–15/16, 27–33/34) were the positive control, and one spot (green colour, 07–17/18) was TRIM21 reacting with anti-IgG antibody. The remaining three red colour spots (09–39/40, 13–09/10, 13–35/36, 15–23/34) and PD-1 spot (15–23/24) were determined as cross-reactive proteins by anti-PD-1 antibody we used.

For the first screening, anti-PD-1 mAb (100 ng/ml, 1/1000 dilution) was applied on a plate for 1 h. After washing, rabbit-IgG-HRP mAb was also applied, and then the plate was treated with detection mixture after washing. On this human protein array, as a positive control, human PD-1 protein was mounted as two spots (02–46 and 02–48 shown as the blue colour on the right-upper area of a plate in Fig. 4A). The first screening results indicated four spots (red colour in Fig. 4B) as positive by imaging (Fig. 4A) and scan data (Fig. 4B). Since the two spots on the right-upper area were human PD-1 protein (a blue ellipse in Fig. 4A), four spots (19–01, 19–46, 19–48, and 25–28 shown as red colour spot in Fig. 4B) were found as positive mixture proteins in the human protein array. Detection of the PD-1 protein indicated that this screening worked well. Next, in the second screening, a total of 170 proteins from four positive and three border spots (17–44, 25–33, and 32–44 shown as black in Fig. 4B), three randomly selected spots (18–39, 26–35, and 31–33), 19 proteins, and control proteins including Venus (27–37/38) and PD-1 were individually spotted in a 1536-well plate as a dual spot. In the plate for the second screening, the PD-1 protein was used as a double dual spot (05–5/16 and 27–33/34) shown in the two blue ellipses in Fig. 4C. The same reaction condition as that of the first screening was used for this second screening. These results from a single image clearly indicated that four dual clones (09–39/40, 13–09/10, 13–35/36, and 15–23/24 shown as red ellipses) and TRIM21 (07–17/18, green ellipse) from the human protein array and two dual positive clones (two blue ellipses) reacted with anti-PD-1 mAb (Table 2). Since TRIM21 has the ability to bind to IgG protein^11,12^ (Supplementary Fig. 2), this was considered background and not cross reactivation. Fortunately, all four positive spots from the first screening had one cross-reactive clone, indicating that the 1536-well formatted CF-PA^2^Vtech is suitable for antigen screening against the antibody.

**Table 2:** Summary of cross-reactive clones by anti-PD-1 antibody.

Clone no.	2nd SC well no.	Entry clone ID	Accession no.	Gene name	Similar epitope region	Similar epitope sequence	Epitope	Mutant name	Mutant sequence
1	9–39/40	FLJ83286WAAF	11181995	hCG2007644	8–16	••LPRRAQRLW••	○	m1	••LAAAAARLW••
					74–82	••CGLSAQYLV	×	m2	••CGLAAAAAV
2	13–9/10	FLJ53560AAAF	AK297376.1	unnamed protein product	247–254	••TPRSAPSS••	×	m1	••TAAAAASS••
					257–263	••FPRSAQK	○	m2	••FAAAAAK
3	13–35/36	FLJ54115AAAF	NM_001257159.1	centrosomal protein of 41 kDa isoform 3	253–261	••GARSAQNLP••	○	m1	••GAAAAAALP••
4	15–23/24	FLJ94476AAAF	NP_005009.2	programmed cell death 1	270–278	••GPRSAQPLR••	○	m	••GAAAAAPLR••

^*^The amino acids identical with PD1 epitope sequence are shown as bold.

### Characterization of three human proteins reacting with anti-PD-1 antibody

**Figure 5:**
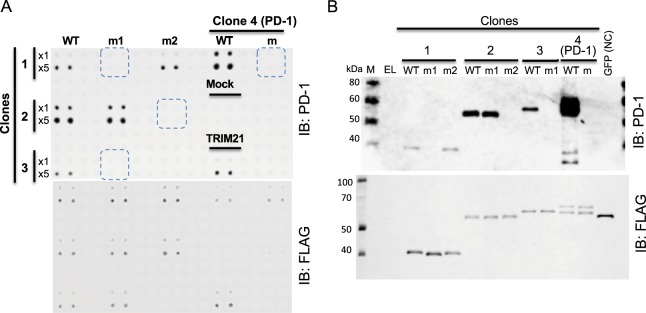
Characterization of cross-reactive antigens by anti-PD-1 antibody. (**A**) The anti-PD-1 antibody cross-reactivity screening using WT and m proteins by CF-PA^2^Vtech. Clone 1 and 2 had the two candidate epitope sequences, and clone 3 had one candidate epitope sequence. These candidate epitope sequences were mutated to alanine residues. WT and m (m, m1, or m2) proteins were synthesized by the cell-free expression system, and then attached to the magnetic beads and immobilized on the array plate in duplicate in different amounts (1×: same protein amount with the first screening array spots, 5×: the five times amount). Arrayed proteins were confirmed with anti-FLAG antibody (lower panel), and cross-reactivity was carried out by anti-PD-1 antibody (upper panel). Loss of cross-reactivity was shown as blue dotted spot area in the upper panel. (**B**) Immunoblotting analysis of the cell-free produced wild and mutant proteins. The same clones, constructions, and antibodies used in (**A**) were used for analysis.

In Fig. 4, CF-PA^2^Vtech using anti-PD-1 mAb indicated cross-reactivity against four human proteins (Table 2). In these four positive proteins, one (clone no. 4 in Table 2) is PD-1 in the human protein array, indicating that validation using anti-PD-1 mAb was working well. Thus, three proteins, FLJ83286WAAF (clone 1), FLJ53560AAAF (clone 2), and FLJ54115 AAAF (clone 3), that were found as cross-reactive clones to anti-PD1 mAb were used. Next, we investigated whether these proteins have a similar epitope recognized by anti-PD-1 antibody (D4W2J). Analysis of sequence similarity were carried out by Harrplot software. Because this antibody was produced by immunizing animals with a synthetic peptide corresponding to residues surrounding Ala274 (•••GPRSA^274^QPLR•••) of human PD-1 protein (from company data sheet), a similar epitope sequence was searched for in these clones. Interestingly, all three proteins have a single or two candidate epitope sequences including SAQ or RxAQ (Table 2 and Supplementary Fig. 4). To confirm whether these sequences were recognized by anti-PD-1 antibody, each epitope candidate was mutated to the alanine sequence. These mutants were analysed by both CF-PA^2^Vtech (Fig. 5A) and immunoblotting (Fig. 5B) using anti-PD-1 mAb. The results showed that three mutants (m1 in clone 1, m2 in clone 2, and m1 in clone 3) and a mutant of PD-1 (clone 4) completely lost the cross-reactivity, indicating that proteins found by CF-PA^2^Vtech have an epitope for anti-PD-1 mAb. In addition, analysis of these mutations revealed that anti-PD-1 mAb (D4W2J) mainly recognizes the RxAQ sequence as an epitope.

## Discussion

In this study, we used both 384- and 1536-well formats for our assay. Using these formats, the required amount of antibody was about 5 µg per plate. Proteins were spotted around 1 µL of translational mixture in a single spot. In total, the amounts of all reagents utilized were realistic. ELISA and AlphaScreen technology are also major candidate methods for antibody validation. However, in general, ELISA is not suitable for use in the screening of around 20,000 kinds of proteins because it requires purified recombinant protein, and washing is a time-consuming step. Large-scale purification of recombinant protein is also very time consuming and requires considerable refining. Because CF-PA^2^Vtech involves carrying out GST-based purification on the magnetic plate, we directly used translational mixtures for spotting proteins. In addition, to identify a single cross-react protein, our approach used the two step screenings of 1st screening and a dual spot of 2nd screening. This denotes that a single cross-react protein was experimentally evaluated by three times, indicating that CF-PA^2^Vtech provides an efficient and reproducible detection system for antibody validation. As one of other methods, AlphaScreen technology is suitable for large-scale validation of antibodies. Previously, we used AlphaScreen technology for antibody validation^13–17^. However, a disadvantage is bead cost; for example, the cost was more than 8,000 US$ for 20,000 assays. CF-PA^2^Vtech is a highly sensitive, specific, and high-throughput analysis method for antibody validation.

We used anti-HA (TANA2) and anti-PD-1 (D4W2J) mAbs in this study. These mAbs are commercially available and high-performed mAbs, because the cross reactivity of these mAbs was very low (Tables 1 and 2). These results indicate the possibility that a huge number of proteins would be obtained as the cross-reactive clones if we used low affinity mAbs or polyclonal antibodies like sera in this method. This means that the use of high-performed mAb is an important point for the antibody validation using CF-PA^2^Vtech. In addition, mAbs used in this study recognized peptides directly. Since proteins synthesized by the wheat cell-free system have not been modified by post-translational modifications such as phosphorylation or glycosylation, our method is not suitable for validation of antibody recognizing these modification regions.

In general, an antibody binding to a protein recognizes a specific epitope consisting of a few amino acid sequences. We have determined epitopes of some mAbs^18–20^. Identification of the epitope, therefore, is very important for understanding the antibody features. However, the epitope mapping of an mAb is very time-consuming and laborious, requiring the construction of many mutants and performing numerous assays step-by-step. In this study, CF-PA^2^Vtech indicated a specific epitope recognized by each mAb, in which anti-HA (TANA2 clone, MBL) and anti-PD-1 (D4W2J clone, CST) mAbs mainly recognized PDY (Table 1) and RxAQ (Table 2), respectively. Because several amino-acid sequences of cross-reactive proteins could be obtained by this assay, a comparison among these sequences provides information about the epitope. This use of a large number of annotated proteins is also a major advantage of this method.

In 19,712 human proteins used, 525 or 74 proteins have PDY or RSAQ sequence respectively. However, our method detected 10 or 3 proteins as the cross-reactive clones of anti-HA (TANA2) or anti-PD-1 (D4W2J) mAb respectively, suggesting that many candidate epitopes are structurally hidden inside these proteins and then could not be recognized by each mAb. These results suggest that antigen proteins in CF-PA^2^Vtech have the folded state. In fact, since proteins produced by the wheat cell-free system have a folding state, autoantibodies recognizing structured epitope reacted to the cell-free synthesized proteins^13^. This therefore suggests that CF-PA^2^Vtech is available for antibodies which recognize structured epitope. In contrast, denaturation of proteins may expose antibody-recognized regions. However, at the same time, the fused GST protein also loses the function under the denatured condition such as high concentration of detergent. Because GST protein is required for capturing target proteins, almost all proteins on plates would be washed out, indicating that the denatured proteins cannot use for CF-PA^2^Vtech.

From sequence analysis, 5,161 proteins in this study have more than a single transmembrane region. CF-PA^2^Vtech using anti-PD-1 antibody could detect PD-1 protein that has a single transmembrane region (Fig. 4). In addition, multiple types of antibodies have reacted each antigen of membrane protein synthesized by the wheat cell-free system in our previous reports^13–18^. These results strongly indicate the potential that CF-PA^2^Vtech would be used for validation of the antibody recognizing membrane protein as antigen. Furthermore, as a production system for the functional membrane proteins, we have developed a liposome-supplemented wheat cell-free translation system^21^ and indicated that proteo-liposome consisting of membrane protein and liposome is very suitable for antigens^18^. For a next generation method, the use of proteo-liposome in CF-PA^2^Vtech may be an important improvement for antibody validation of membrane protein.

The 2018 Nobel prize in physiology or medicine was awarded to those responsible for the “discovery of cancer therapy by inhibition of negative immune regulation”. As indicated by this recent prize, the importance of antibody drugs is increasing yearly, because antibodies potentially have high affinity and specificity against specific proteins. However, all antibodies do not have these abilities. Therefore antibody validation is a key step for identification of high-performance antibodies. In particular, for antibody drugs, the cross-reactivity of the antibody is a very important issue^1^. Because CF-PA^2^Vtech can validate the cross-reactivity of antibodies using ~20,000 human proteins, we are convinced that our assay system could be useful in such studies.

## Materials and Methods

### Antibodies

The antibodies used in this study are listed as follows: Anti-HA-tag mAb (1/5000 dilution) [HRP-DirecT (mouse monoclonal TANA2 clone, MBL)], Anti-DDDDK-Alexa Fluor 647 (1/5000 dilution, MBL, M185-A64), anti-PD-1 antibody [PD-1 (D4W2J) XP Rabbit mAb (1/1000 dilution), CST (#86163)], mouse anti-FLAG M2-HRP mAb (1/10000 dilutiomn, SIGMA, #A8592), and anti-rabbit IgG-HRP (1/5000 dilution) (NA934, GE Healthcare).

### Construction of the *in vitro* transcription templates

Each gene was selected from the cDNA library from the Functional Analysis of Protein and Research Application Project (NEDO, FLJ Human cDNA Database, http://flj.lifesciencedb.jp)^10^ and then was amplified by PCR. The DNA fragments of the open-reading frame (ORF) were cloned in a pDONR201 vector using the gateway cloning system (Thermo Fisher Scientific). After confirming sequences, we generated these expression vectors by LR Clonase recombination with pEU-FLAG-GST-GW vectors for *in vitro* transcription. Then, these regions of the gene containing the ORF and tag sequence were amplified by PCR and used as transcription templates.

### Wheat cell-free protein synthesis

For construction of the protein array, we made a new method that a protein was synthesized in a well on 384-well plate (Supplementary Fig. 4). The FLAG-GST fusion human full-length proteins were synthesized by using the WEPRO7240G Expression Kit (CellFree Sciences, Matsuyama, Japan) as follows: translation reactions were performed using a bilayer method^22^. For the bilayer system, 45.58 µl of SUB-AMIX SGC (CellFree Sciences) was overlaid with 4.42 µl of reaction mixture containing 1.67 µl of WEPRO7240G wheat germ extract, 0.11 µl RNase inhibitor, 2.5 µl of mRNA, and 0.14 µl of 20 mg/ml creatine kinase in a 384well titre-plate and incubated at 26°C for 18 h. All dispensing processes for protein synthesis were carried out by a fully automatic dispenser (HTS10-HD, FUJIFILM Wako Pure Chemical Corporation).

Synthesis of selected proteins after validation using CF-PA^2^Vtech was performed by using the bilayer method via the WEPRO7240G Expression Kit (CellFree Sciences) according to the manufacturer’s instructions in a 384-well plate (Supplementary Fig. 4). Expression of these proteins has been indicated in web site of Human Gene and Protein Database (HGPD, http://hgpd.lifesciencedb.jp/cgi/index.cgi).

### Construction of human protein array

Protein synthesis of 19,712 human full-length cDNA harbouring FLAG and GST (FG) tags was performed with the wheat germ protein expression system described above, and the synthesized proteins were absorbed onto the surface of glutathione-coupled magnetic beads on an array plate (384 wells × 12 or 1,536 wells × 1 plate, CF-PA^2^Vtech, CellFree Science, Matsuyama, Japan), as follows. Magnetic beads with glutathione ligand were added to a reaction mixture containing a synthesized protein bearing the FG tag, and the synthesized protein was absorbed onto the surface of the beads. Beads with adsorbed synthesized protein were dispensed on the array plate, which had a magnetic plate at the bottom. Each human protein was immobilized at the bottom of the array plate via the magnetic beads in the solution.

### Antibody validation using human protein array

The arrays were blocked with 50 mM HEPES (pH 7.5), 200 mM NaCl, 0.08% Triton-X, 25% Glycerol, 5 mM GSH, and 0.3% skim milk.

For anti-HA antibody validation, the arrays were incubated with anti-HA antibody (1/5,000 dilution) in TBS buffer containing 1% skim milk and 0.04% Brij-35 at 25 °C for 1 h. After extensive washing with TBS-0.04% Brij 35, a chemiluminescence reagent (ImmunoStar^®^ LD, FUJIFILM Wako Pure Chemical Corporation, Osaka, Japan) was used for detection of binding signals using ImageQuant LAS4000 (GE Healthcare UK Ltd.). Array-Pro Analyzer (Nippon Roper Ltd.) was used for signal quantification. For analysis of sequence similarity, we used Harrplot analysis, one of Genetyx(GENETYX Corp., Tokyo, Japan)‘s applications.

For anti-PD-1 antibody validation, the arrays were incubated with anti-PD-1 antibody (100 ng/ml) in PBS buffer containing 1x Synthetic block (Thermo Fisher Scientific Corp., Carlsbad, CA, USA) at 25 °C for 1 h. After extensive washing with TBST, the arrays were incubated with horseradish peroxidase (HRP)-conjugated anti-rabbit IgG (1/5,000 dilution) in PBS buffer containing 1x Synthetic block at room temperature (about 25 °C) for 1 h. After extensive washing with TBST, a chemiluminescence reagent was used for detection of binding signals using ImageQuant LAS4000. Array-Pro Analyzer was used for signal quantification. For analysis of sequence similarity, we used Harrplot analysis.

### Cell transfections

Each selected gene was inserted into pcDNA3.1-FLAG plasmid. HEK293T cells were incubated at 37 °C with 5% CO_2_ in DMEM (Dulbecco’s modified Eagle’s medium; Low Glucose) with L-Glutamine and Phenol Red (Wako) with 10% Fetal Bovine Serum Ireland Origin Sterile Filtered (biosera) and antibiotics (100 units/mL penicillin and 100 μg/mL streptomycin) (GIBCO). HEK293T cells were transfected with plasmids using Opti-MEM I (1×) Reduced Serum Medium and PEI MAX - Transfection Grade Linear Polyethylenimine Hydrochloride (Polysciences).

### Immunoblotting

Immunoblotting was carried out following standard protocols. Briefly, proteins were applied to SDS-polyacrylamide gel electrophoresis (SDS-PAGE) and transferred onto a PVDF membrane by wet blotting. After blocking with 5% milk/TBST, the membrane was tested using the indicated antibodies and a horseradish peroxidase (HRP)-conjugated antibody or alexa fluor 647 labeled antibody. All uncropped blot images are provided in Supplementary Figs. 5 and 6.

## Supplementary information

Supplementary Information

## References

[1] Peng HP, Lee KH, Jian JW, Yang AS (2014). Origins of specificity and affinity in antibody-protein interactions. Proc Natl Acad Sci USA.

[2] Kaplon H, Reichert JM (2019). Antibodies to watch in 2019. MAbs.

[3] de Taeye SW, Rispens T, Vidarsson G (2019). The Ligands for Human IgG and Their Effector Functions. Antibodies.

[4] Ohaegbulam KC, Assal A, Lazar-Molnar E, Yao Y, Zang X (2015). Human cancer immunotherapy with antibodies to the PD-1 and PD-L1 pathway. Trends Mol Med.

[5] Sharpe AH, Pauken KE (2018). The diverse functions of the PD1 inhibitory pathway. Nat Rev Immunol.

[6] Chan CE, Chan AH, Hanson BJ, Ooi EE (2009). The use of antibodies in the treatment of infectious diseases. Singapore Med J.

[7] Salazar G, Zhang N, Fu TM, An Z (2017). Antibody therapies for the prevention and treatment of viral infections. NPJ Vaccines.

[8] Sparrow E, Friede M, Sheikh M, Torvaldsen S (2017). Therapeutic antibodies for infectious diseases. Bull World Health Organ.

[9] Bordeaux J (2010). Antibody validation. Biotechniques.

[10] Goshima N (2008). Human protein factory for converting the transcriptome into an *in vitro*-expressed proteome. Nat Methods.

[11] Mallery DL (2010). Antibodies mediate intracellular immunity through tripartite motif-containing 21 (TRIM21). Proc Natl Acad Sci USA.

[12] Rhodes DA, Isenberg DA (2017). TRIM21 and the function of antibodies inside cells. Trends Immunol.

[13] Matsuoka K, Komori H, Nose M, Endo Y, Sawasaki T (2010). Simple screening method for autoantigen proteins using the N-terminal biotinylated protein library produced by wheat cell-free synthesis. J Proteome Res.

[14] Furukawa H (2015). Autoantibody Profiles in Collagen Disease Patients with Interstitial Lung Disease (ILD): Antibodies to Major Histocompatibility Complex Class I-Related Chain A (MICA) as Markers of ILD. Biomark Insights.

[15] Onishi S (2015). Novel Autoantigens Associated with Lupus Nephritis. PLoS One.

[16] Ishigami T (2013). Anti-interleukin-5 and multiple autoantibodies are associated with human atherosclerotic diseases and serum interleukin-5 levels. FASEB J.

[17] Mizutani Y (2013). Novel approach to identifying autoantibodies in rheumatoid synovitis with a biotinylated human autoantigen library and the enzyme-labeled antigen method. J Immunol Methods.

[18] Takeda H (2015). Production of monoclonal antibodies against GPCR using cell-free synthesized GPCR antigen and biotinylated liposome-based interaction assay. Sci Rep.

[19] Yano T (2016). AGIA Tag System Based on a High Affinity Rabbit Monoclonal Antibody against Human Dopamine Receptor D1 for Protein Analysis. PLoS One.

[20] Takeda H (2017). CP5 system, for simple and highly efficient protein purification with a C-terminal designed mini tag. PLoS One.

[21] Nozawa A (2011). Production and partial purification of membrane proteins using a liposome-supplemented wheat cell-free translation system. BMC Biotechnol.

[22] Sawasaki T (2002). A bilayer cell-free protein synthesis system for high-throughput screening of gene products. FEBS Lett.

