# Supplementary material for: CF-PA2Vtech: a cell-free human protein array technology for antibody validation against human proteins
Source: Sci Rep. 2019 Dec 18;9:19349. doi: 10.1038/s41598-019-55785-5 (PMC6920144; doi:10.1038/s41598-019-55785-5)
Supplement: Supplementary file 1 — Supplementary Information [file 41598_2019_55785_MOESM1_ESM.pdf]

Supplemental Information

CF-PA^{2}Vtech: a cell-free human protein array technology for antibody validation against human proteins

Ryo Morishita[1*], Shusei Sugiyama[1], Miwako Denda[1], Soh Tokunaga[2], Kohki Kido[2], Ryouhei Shioya[2], Satoshi Ozawa[1], and Tatsuya Sawasaki[2*]

[1]*CellFree Sciences. Co. Ltd., 3 Bunkyo-cho, Matsuyama, Ehime 790-8577, Japan,*
[2]*Proteo-Science Center, 3 Bunkyo-cho, Matsuyama, Ehime 790-8577, Japan*

*Corresponding Authors:

Ryo Morishita (CellFree Sciences. Co. Ltd., Matsuyama 790-8577, Japan)
E-mail: rmorishita@cfsciences.com

Tatsuya Sawasaki (Proteo-Science Center, Ehime University, Matsuyama 790-8577, Japan)
E-mail: sawasaki@ehime-u.ac.jp.

Supplementary Figures 1-6

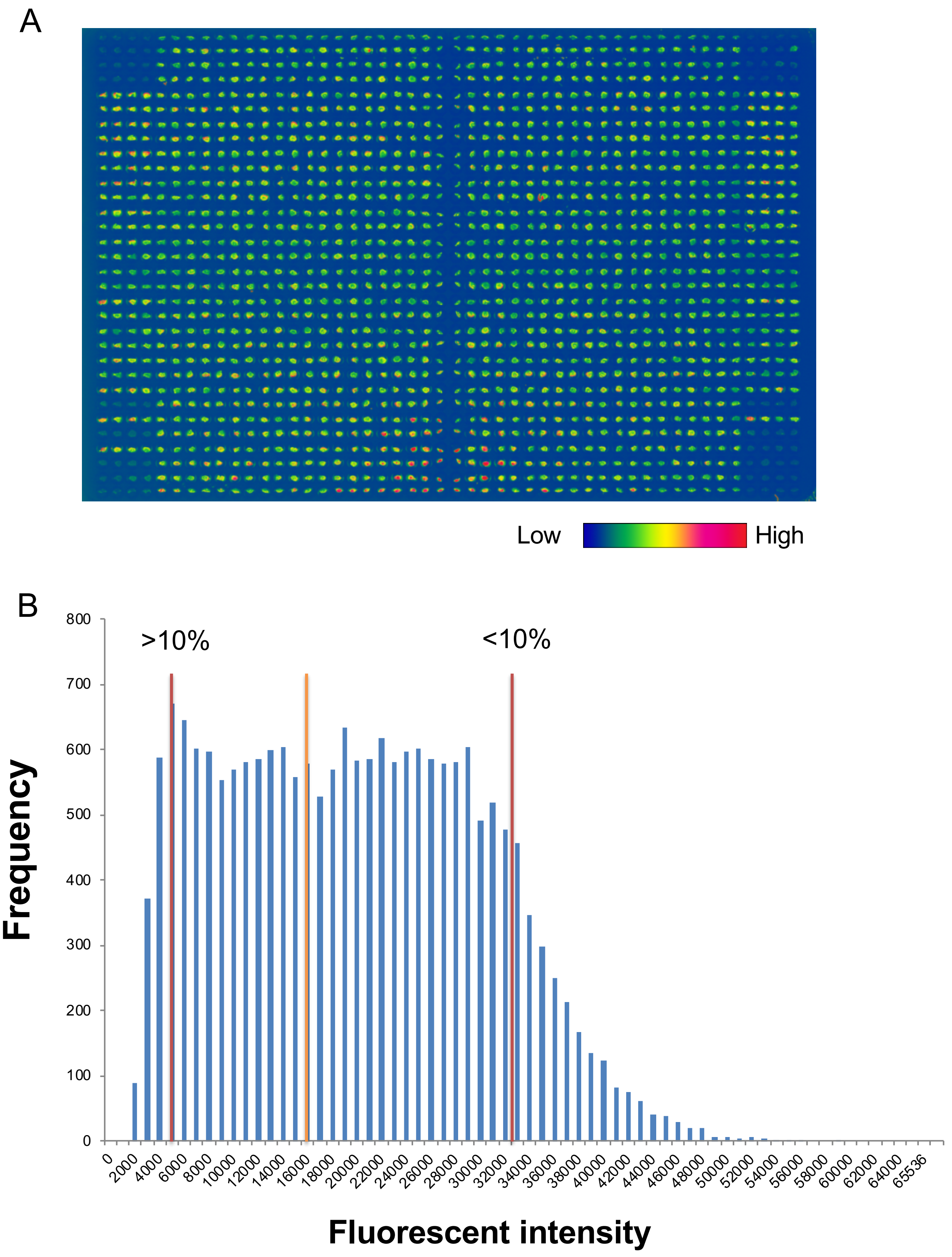

Supplementary Figure 1. Frequency of fluorescent intensity on 1536-well format.

Arrayed proteins were detected using a fluorescence-labeled anti-FLAG antibody, and the diversity of fluorescence intensity of each arrayed protein was investigated. Typhoon FLA 9500 (GE Healthcare UK Ltd.) was used for fluorescence detection. Array-Pro Analyzer was used for signal quantification. Fluorescence intensity showed that more than 80% of wells were found within the high signal zone.

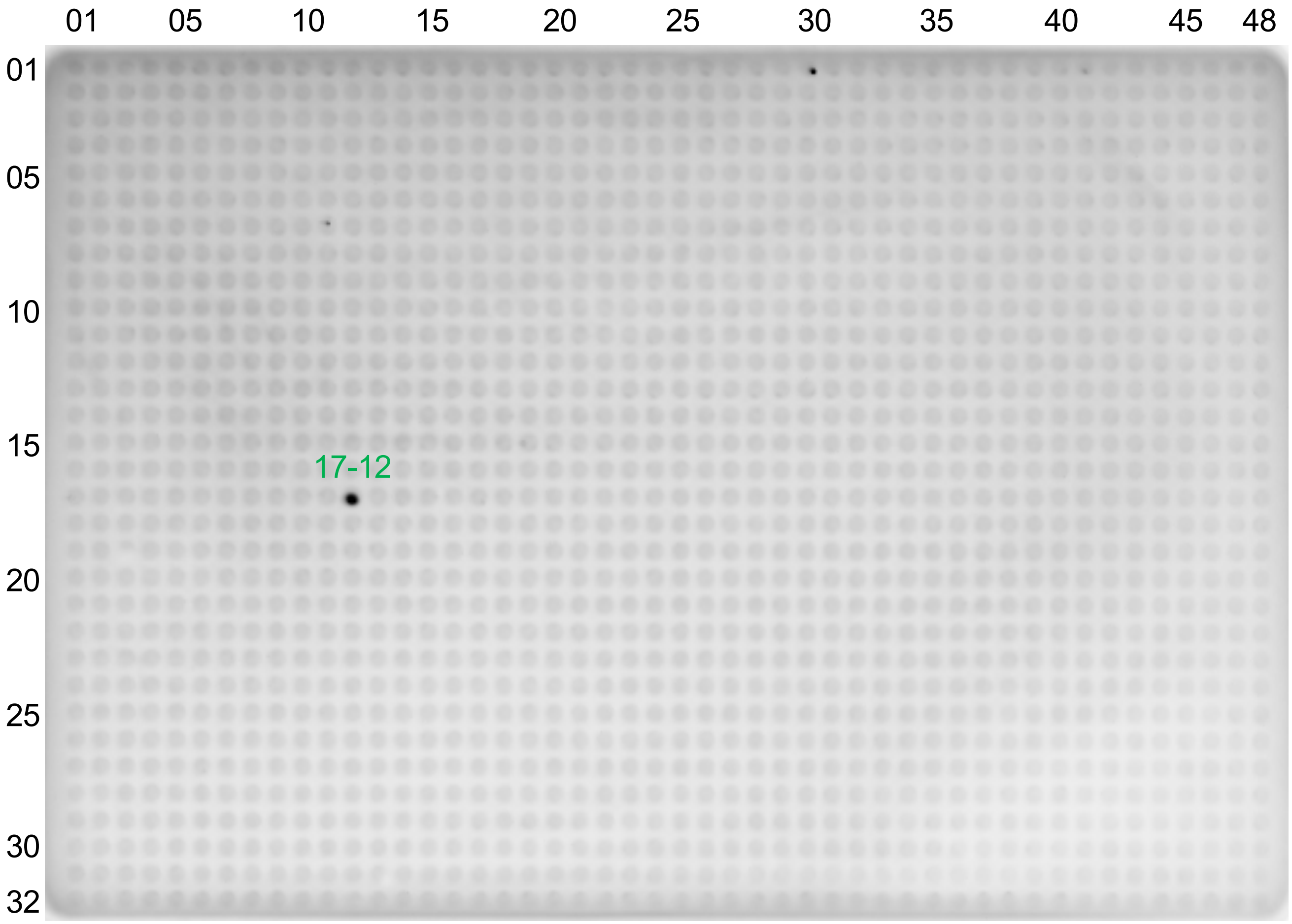

Supplementary Figure 2. Results of reaction by anti-rabbit IgG antibody on a single 1536-well format.

Single spot (17-12) showed cross-reactivity against anti-rabbit IgG antibody.

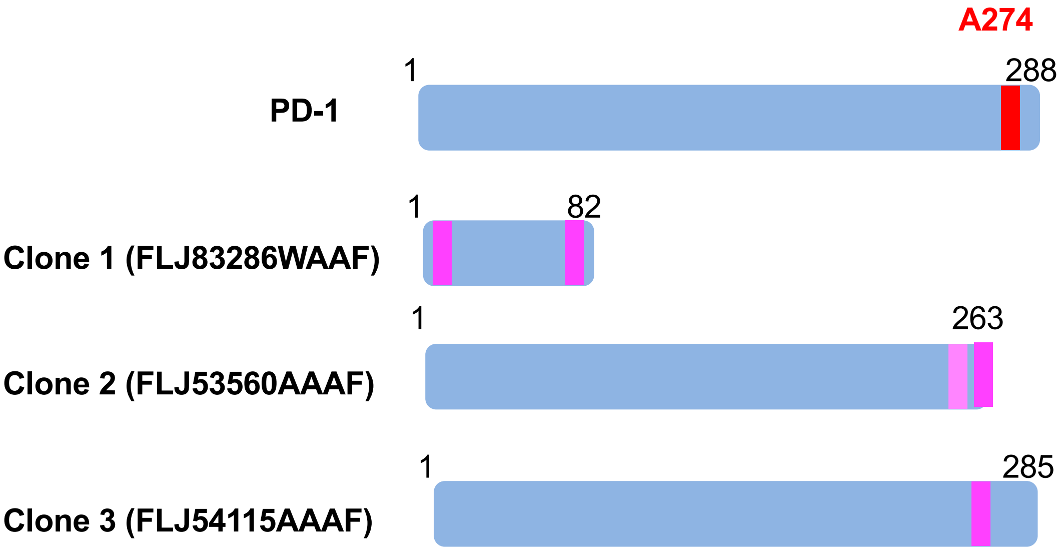

Supplementary Figure 3. Location of similar epitope sequences in cross-reactive proteins using anti-PD-1 antibody.

Clone 1 (FLJ83286WAAF) and Clone 2 (FLJ53560AAAF), or Clone 3 (FLJ54115AAAF) have two or one different candidate epitope sequences, respectively. The candidate epitope is shown as the pink colour bar. Red colour bar in PD-1 protein denotes a position of antigen peptide of anti-PD-1 antibody used.

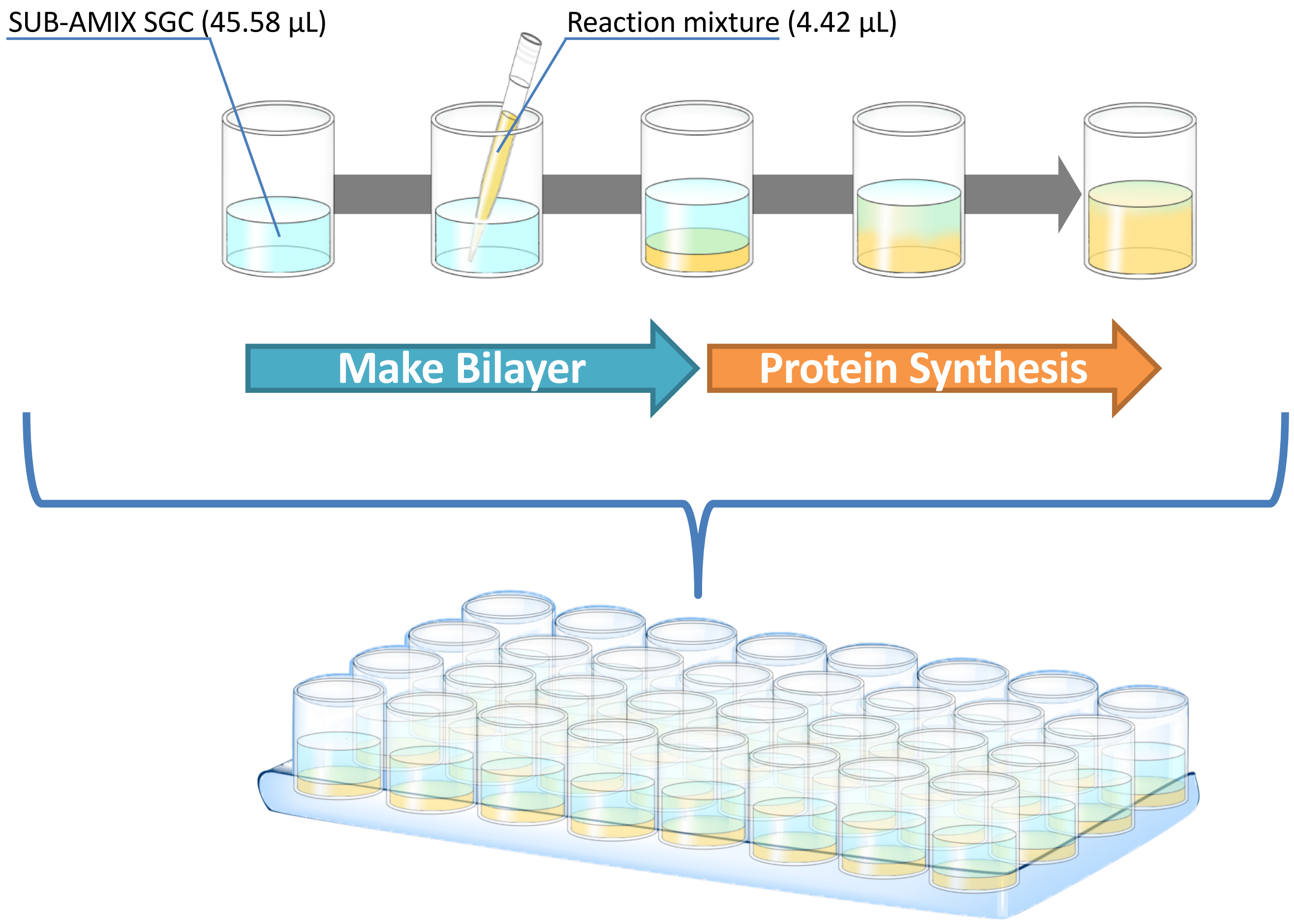

Supplementary Figure 4. Protein synthesis scheme using a wheat cell-free protein synthesis on 384-well plate.

A protein was synthesized in a well on 384-well plate. All dispensing processes for protein synthesis were carried out by a fully automatic dispenser HTS10-HD.

Figure 3A

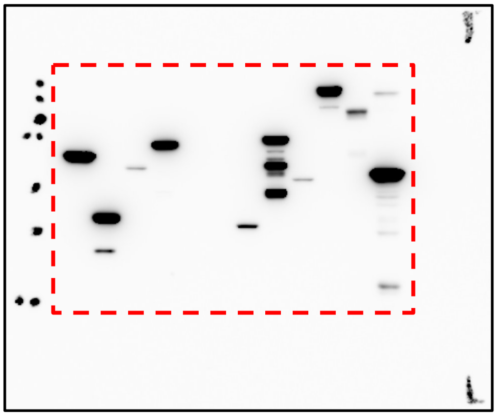

IB: HA

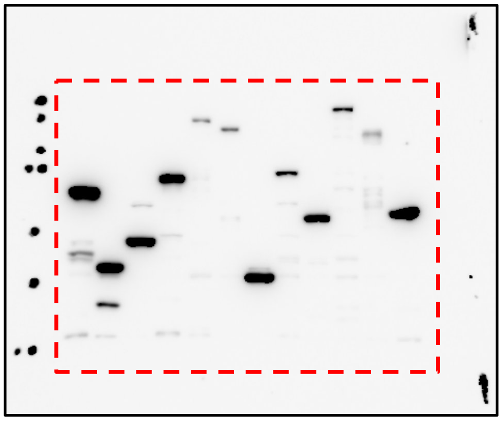

IB: FLAG

Figure 3B

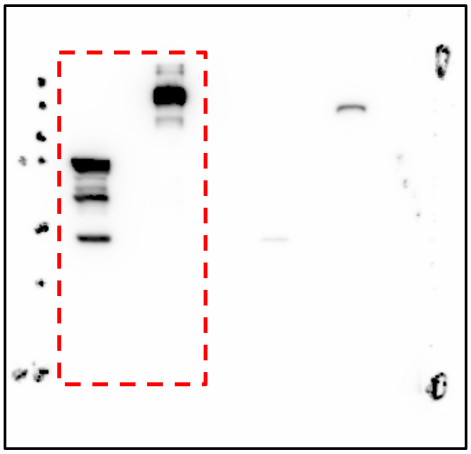

IB: HA

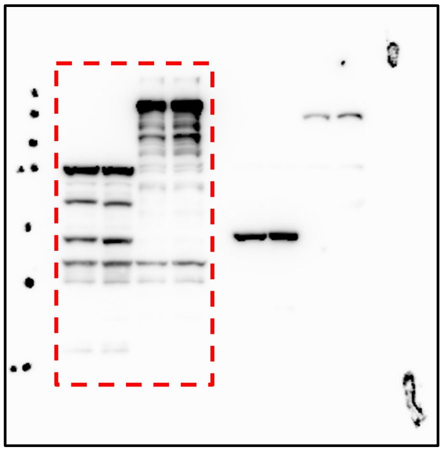

IB: FLAG

Figure 3C

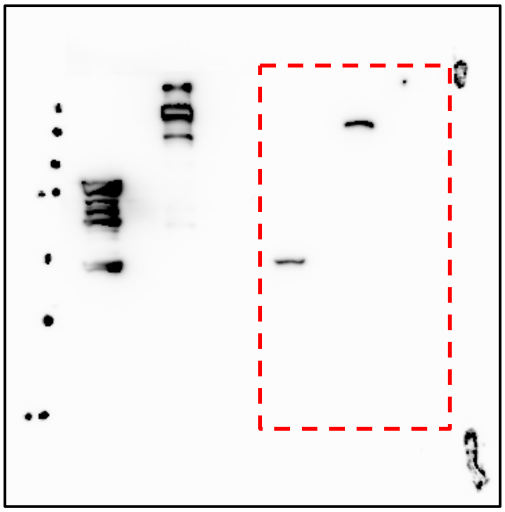

IB: HA

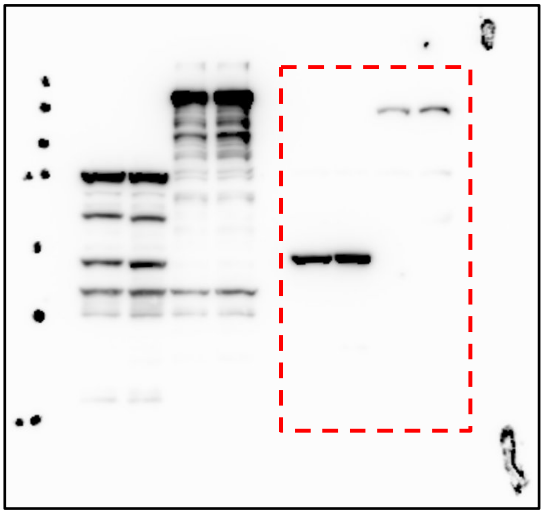

IB: FLAG

Supplementary Figure 5. Uncropped immunoblots in Figure 3.

Figure 5B

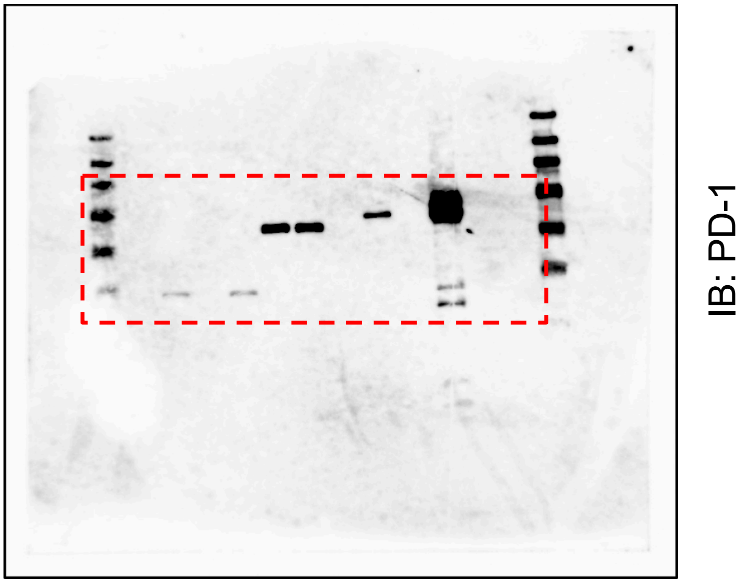

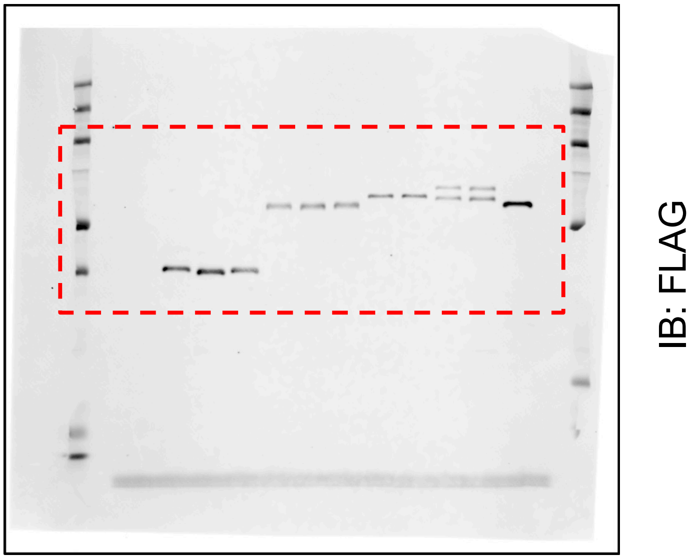

Supplementary Figure 6. Uncropped immunoblots in Figure 5.
